# Cardiovascular disease risk factors and socioeconomic variables in a nation undergoing epidemiologic transition

**DOI:** 10.1186/1471-2458-13-886

**Published:** 2013-09-25

**Authors:** Rajah Rasiah, Khalid Yusoff, Amiri Mohammadreza, Rishya Manikam, Makmor Tumin, Sankara Kumar Chandrasekaran, Shabnam Khademi, Najmin Abu Bakar

**Affiliations:** 1 Faculty of Economics and Administration, University of Malaya, 50603 Kuala Lumpur, Malaysia; 2 Faculty of Medicine, Universiti Teknologi Mara, Shah Alam 40450, Selangor, Malaysia; 3 Faculty of Medicine, University of Malaya, 50603 Kuala Lumpur, Malaysia

**Keywords:** Cardiovascular disease risk factors, Education, Income, Malaysia

## Abstract

**Background:**

Cardiovascular disease (CVD) related deaths is not only the prime cause of mortality in the world, it has also continued to increase in the low and middle income countries. Hence, this study examines the relationship between CVD risk factors and socioeconomic variables in Malaysia, which is a rapidly growing middle income nation undergoing epidemiologic transition.

**Methods:**

Using data from 11,959 adults aged 30 years and above, and living in urban and rural areas between 2007 and 2010, this study attempts to examine the prevalence of CVD risk factors, and the association between these factors, and socioeconomic and demographic variables in Malaysia. The socioeconomic and demographic, and anthropometric data was obtained with blood pressure and fasting venous blood for glucose and lipids through a community-based survey.

**Results:**

The association between CVD risk factors, and education and income was mixed. There was a negative association between smoking and hypertension, and education and income. The association between diabetes, hypercholesterolemia and being overweight with education and income was not clear. More men than women smoked in all education and income groups. The remaining consistent results show that the relationship between smoking, and education and income was obvious and inverse among Malays, others, rural women, Western Peninsular Malaysia (WPM) and Eastern Peninsular Malaysia (EPM). Urban men showed higher prevalence of being overweight than rural men in all education and income categories. Except for those with no education more rural men smoked than urban men. Also, Malay men in all education and income categories showed the highest prevalence of smoking among the ethnic groups.

**Conclusions:**

The association between CVD risk factors and socioeconomic variables should be considered when formulating programmes to reduce morbidity and mortality rates in low and middle income countries. While general awareness programmes should be targeted at all, specific ones should be focused on vulnerable groups, such as, men and rural inhabitants for smoking, Malays for hypertension and hypercholesterolemia, and Indians and Malays, and respondents from EPM for diabetes.

## Background

Cardiovascular disease (CVD) is the main cause of death in the world [1-5]. However, whereas the adult CVD death rate in developed economies has declined since the 1970s [1] it has risen in the low and middle income countries [1]. Low and middle income countries contributed the highest percentage of CVD deaths worldwide, which rose from 14.4 million in 1990 to 16.5 million in 2005 [1]. In Malaysia, which was ranked as a middle income country in 2010 [6], in-hospital CVD deaths shot up from 15.7% in 2006 to 25.4% in 2009 [7,8]. The prevalence of CVD risk factors in Malaysia have also risen: the rates of diabetes, hypertension and hypercholesterolemia rose from 8.3%, 33.0% and 5.0% respectively, in 1996 to 14.9%, 42.6% and 24.0% respectively, in 2006 [7,8].

Data from the developed countries show an inverse relationship between socio-economic status (SES), and CVD risk factors of smoking [9-13], high blood pressure [11,14-17] and overweight [11,13,14]. However, past findings on Malaysia are mixed [18-24]. This study provides current evidence on the relationship between prevalence of CVD risk factors and SES variables in Malaysia. In addition, this study for the first time examines the respondents by three regional classifications, Western Peninsular Malaysia (WPM), Eastern Peninsular Malaysia (EPM and East Malaysia (EM). The advantage of this study over past studies on multiple CVD risk factors on Malaysia are, one, the sample is much larger than many other studies [21,22], two, it has a much wider geographical coverage than many other studies [19,25-27], and three, it examines more CVD risk factors than several studies [25,26,28,29].

## Methods

The prevalence of CVD risk factors was derived from a community-based health survey on 11,959 adult volunteers (aged ≥ 30 years) conducted by the REDISCOVER Study team in 2007–2010. Of this number we were able to obtain complete income data only from 7,135 respondents, and hence, the analysis is based on this number. Part of the data contributed to the Prospective Urban Rural Epidemiological (PURE) study [30,31]. Volunteers were invited to attend community centres in a fasting state where demographic and anthropometric data and blood pressures, as well as, venous blood for glucose and lipid readings were screened and recorded. The community centres were from Selangor (5), Kuala Lumpur (3) and Negri Sembilan (2), all of which are urbanized and located in WPM, and from the EPM states of Pahang (4) and Kelantan (4), and the EM state of Sabah (1). The respondents gave a written consent to participate at recruitment into the study, which was approved by the institutional research ethics committee. To attract significant participation in the community survey, the REDISCOVER team pledged to monitor their CVD risk factors over the period 2007–2016. It is for these reasons the ethnic breakdown of the sample of 71.4% Malays, 10.5% Chinese, 2.9% Indians and 15.2% others (includes non-Malay natives and other Malaysians not classified among Malays, Chinese and Indians) differs from the ethnic breakdown of the 2010 national population of, 55.1% Malays, 24.6% Chinese, 7.3% Indians and 13.0% others [32]. Given that the survey involves health screening during the study, and the large size of the sample, we believe the sample is sufficiently robust for meaningful interpretation of the results. In doing so we followed the same sampling procedure used by Yusof et al. [30].

The prevalence rates were age-adjusted using 2010 standard Malaysian population, and income was inflation adjusted using the consumer price index for the years 2007–2010. We used the jack-knifed approach by writing a module in SPSS 20 to avert biased estimates of standard errors (SEs) [33-35]. Weighted standard deviation was employed to derive the distribution of the variables. The number of observations of each CVD risk factors varies because of missing responses.

Hypercholesterolemia is defined as fasting total plasma cholesterol of ≥ 5.2 mmol/L [10], regular smokers if they smoked at least one cigarette a day [11], and hypertensive if their blood pressures were ≥ 140/90 mmHg or were on anti-hypertensive medications or aware of being hypertensive [11,17,36,37]. Blood pressure was taken twice, and the mean value was used. The measurement of blood pressure was standardized across the sample. A body mass index (BMI, kg/m^2^) of 25.0 or more was considered as overweight [38-40]. Diabetes is diagnosed when fasting glucose was ≥ 126 mg/dL (or 7.0 mmol/L) [41,42]. Although the BMI thresholds vary with different ethnic groups [41], we did not take different BMI thresholds for each of them because it would complicate the inter-ethnic statistical comparison, and also because past studies on Malaysia have used the same thresholds as defined by the Ministry of Health of Malaysia [25,26,28,29].

Education was grouped into the categories of no education, primary, secondary, technical and university education. Income levels were classified as low, middle and high according to annual incomes of ≤ MYR10,000, >MYR10,000-MYR50,000, and > MYR50,000 per annum [43,44]. Income was not adjusted for inflation because of the low increase in the consumer price index in Malaysia during the period 2007–2010. The participants were also classified according to the age-groups of 30–39, 40–49, 50–59, 60–69, ≥70 to obtain the most efficient jackknifed estimates. Ethnically, the participants were grouped into Malays, Chinese, Indians and others. The WPM states were more urbanized than EPM and EM [43,45]. We excluded the technically educated when examining the relationship between CVD risk factors and education because they are of the same level as secondary education. While the sample details of each CVD risk factor are shown in Table 1, the sample details by education and income are shown in Tables 2 and 3.

**Table 1:** Number of men and women in sample by CVD risk factors

**Risk factors**	**Responses**	**Men**	**Women**
Hypertension*	No	2728	3969
	Yes	2509	2753
**Diabetes+**	No	4157	5600
	Yes	1080	1122
**Hypercholesterolemiaδ**	No	1684	2092
	Yes	3553	4630
**Regular Smokerθ**	No	2451	5985
	Yes	2789	737
**Overweightψ**	No	3752	4400
	Yes	1485	2322

Note:

*Mean *SBP* (Systolic Blood Pressure) ≥ 140 mm Hg or Mean *DBP* (Diastolic Blood Pressure) ≥ 90 mm Hg or using medication.

**+**Mean plasma glucose ≥ 126 mg/dL or 7.0 mmol/Lit.

δMean Plasma cholesterol ≥ 5.2 mmol/Lit.

θSmokes at least one cigarette per day.

ψ*BMI* (Body Mass Index) ≥ 25.0.

**Table 2:** Number of men and women in sample by education level, gender, age, ethnicity and location

**Age/Ethnicity/**	**Men**					**Women**				
**Location/Region**	**University**	**Technical**	**Secondary**	**Primary**	**None**	**University**	**Technical**	**Secondary**	**Primary**	**None**
**30 – 39**	136	23	190	35	7	191	14	403	101	37
**40 – 49**	325	49	584	191	68	389	27	984	357	138
**50 – 59**	323	35	581	451	122	269	20	711	578	332
**60 – 69**	146	12	280	451	155	50	9	204	405	336
**≥ 70**	16	9	62	171	179	7	10	22	85	250
**Malay**	725	108	1305	1015	266	710	45	1835	1138	572
**Chinese**	171	17	190	58	24	185	28	274	99	54
**Indian**	51	7	52	14	9	53	4	66	25	17
**Others**	23	3	173	214	227	25	4	191	263	447
**Urban**	867	103	961	357	112	867	64	1361	452	219
**Rural**	104	32	760	942	422	107	17	1010	1072	876
**WPM**	616	92	1017	580	224	559	45	1365	699	518
**EPM**	272	35	551	496	216	145	16	504	440	434
**EM**	83	8	153	225	94	270	20	502	389	1689

**Table 3:** Number of men and women in sample by income level, gender, age, ethnicity and location

**Age/Ethnicity/**	**Men**			**Women**		
**Location/Region**	**Low**	**Middle**	**High**	**Low**	**Middle**	**High**
**30 – 39**	**105**	**125**	**45**	**225**	**159**	**59**
**40 – 49**	**430**	**415**	**153**	**621**	**418**	**207**
**50 – 59**	**586**	**345**	**179**	**698**	**271**	**154**
**60 – 69**	**585**	**150**	**37**	**442**	**84**	**25**
**≥ 70**	**152**	**17**	**3**	**87**	**7**	**10**
**Malay**	**1379**	**890**	**305**	**1463**	**758**	**345**
**Chinese**	**26**	**70**	**78**	**23**	**98**	**68**
**Indian**	**9**	**38**	**28**	**17**	**37**	**26**
**Others**	**448**	**57**	**8**	**574**	**54**	**5**
**Urban**	**393**	**648**	**383**	**433**	**612**	**419**
**Rural**	**1474**	**408**	**36**	**1652**	**336**	**26**
**WPM**	**987**	**667**	**280**	**897**	**508**	**245**
**EPM**	**590**	**269**	**90**	**622**	**176**	**43**
**EM**	**290**	**120**	**49**	**566**	**264**	**157**

## Results and discussion

The association between CVD risk factors, and education and income was clear only with smoking and hypertension, and it was negative, which is consistent with most findings [9-12,21,22]. Also, more men smoked than women in all education and income categories, which is consistent with the findings on Malaysia [21,22]. The relationship between hypertension and age was positive among women in all education groups, but was only positive among men in the university, secondary and primary groups. Also, there was a positive association between hypertension and age among men and women in all income groups.

### Education

The prevalence of CVD risk factors by education varied considerably (Table 4). In both sexes, the prevalence of hypertension was highest among those without education (men 60%, p < 0.001; women 56%, p < 0.001) followed by primary education (men 58%, p < 0.001; women 53%, p < 0.001), which is consistent with some findings on Malaysia [21,22]. The prevalence of diabetes in women was lower than in men. Men had higher prevalence of diabetes than women among the university, technical and secondary educated. The prevalence was similar among those with primary education, while the no education grouped showed the highest prevalence. Men showed higher prevalence of hypercholesterolemia than women in the university and technical education groups. Whereas the prevalence among men and women was the same in the secondary education group, women showed higher prevalence among the primary and no education groups. Among men, the prevalence of hypercholesterolemia was highest in the secondary education (71%, p < 0.001) group and lowest in the no education (53%, p < 0.001) group. The prevalence of smoking was high among men in all categories, which is consistent with the findings from several countries [23,24,46-48]. The highest prevalence of smoking was found among men with primary or no education. The prevalence of being overweight among men was highest in the secondary (34%, p < 0.001), technical (34%, p < 0.001) and university education (34%, p < 0.001) groups. It was lowest among men with no education followed by primary education. The prevalence of being overweight among women was highest in the secondary (42%, p < 0.001) followed by the primary (35%, p < 0.001) education groups.

**Table 4:** Age-adjusted percentage of men and women having CVD risk factors by education level

**CVD risk factors**	**Men**					**Women**				
	**University**	**Technical**	**Secondary**	**Primary**	**None**	**University**	**Technical**	**Secondary**	**Primary**	**None**
Hypertension*	**42 (0.001)**	**40 (0.001)**	**47 (0.001)**	**58 (0.001)**	**60 (0.001)**	**27 (0.001)**	**31 (0.001)**	**39 (0.001)**	**53 (0.001)**	**56 (0.001)**
**Diabetes+**	**16 (0.001)**	**18 (0.001)**	**22 (0.001)**	**20 (0.001)**	**15 (0.001)**	**9 (0.001)**	**8 (0.01)**	**17 (0.001)**	**20 (0.001)**	**17 (0.001)**
**Hypercholesterolemiaδ**	**69 (0.001)**	**70 (0.001)**	**71 (0.001)**	**69 (0.001)**	**53 (0.001)**	**68 (0.001)**	**63 (0.001)**	**71 (0.001)**	**74 (0.001)**	**68 (0.001)**
**Smokerθ**	**42 (0.001)**	**52 (0.001)**	**48 (0.001)**	**55 (0.001)**	**55 (0.001)**	**3 (0.001)**	**6 (0.05)**	**4 (0.001)**	**6 (0.001)**	**16 (0.001)**
**Overweightψ**	**34 (0.001)**	**34 (0.001)**	**34 (0.001)**	**24 (0.001)**	**13 (0.001)**	**35 (0.001)**	**18 (0.001)**	**42 (0.001)**	**41 (0.001)**	**19 (0.001)**

Note: Figures in parentheses refer to p-values.

*Mean *SBP* (Systolic Blood Pressure) ≥ 140 mm Hg or Mean *DBP* (Diastolic Blood Pressure) ≥ 90 mm Hg or using medication

**+**Mean plasma glucose ≥ 126 mg/dL or 7.0 mmol/Lit;

δMean Plasma cholesterol ≥ 5.2 mmol/Lit;

θSmokes at least one cigarette per day;

ψ*BMI* (Body Mass Index) ≥ 25.0.

The prevalence of smoking in men was significantly higher than women in all education groups (Table 5). Also, the less educated groups showed higher prevalence of smoking than the most educated group, which corroborated with previous findings on Malaysia [23,24]. The highest prevalence of smoking was found among men with technical education in the age group of 30–39 (83%, p < 0.001). However, the relationship between smoking and education by ethnicity was only obvious among Malay and other ethnic groups, and it was inverse. Malay men showed the highest prevalence of smoking in all education groups, which corroborated with previous study [21]. Malays with technical education showed the highest prevalence (45%, p < 0.001). The relationship between smoking and education was negative among rural women. Except for respondents with no education, rural men showed higher prevalence of smoking than urban men. Also, rural women showed either higher than or equal prevalence with urban women in all education categories. The relationship between smoking and education was only clear among women in WPM and EPM, and it was inverse. Also, there was not much difference in the prevalence of smoking among the three regions in men with university, secondary and primary education. However, the prevalence of smoking was much higher among men in WPM (58%, p < 0.001) and EPM (57%, p < 0.001) than men in EM (25%, p < 0.1) in the technical education group. Whereas there was not much difference in the prevalence levels of women with university, secondary and primary education in the three regions, EPM showed significantly higher prevalence levels than the other regions among women with technical (13%, p < 0.001) and no education (17%, p < 0.001).

**Table 5:** Age-adjusted percentage of men and women smoking by education, gender, ethnicity and location

**Age/Ethnicity/**	**Men**					**Women**				
**Location/Region**	**University**	**Technical**	**Secondary**	**Primary**	**None**	**University**	**Technical**	**Secondary**	**Primary**	**None**
**30 – 39**	**48 (0.001)**	**83 (0.001)**	**62 (0.001)**	**63 (0.001)**	**57 (0.05)**	**3 (0.001)**	**7 (0.1)**	**4 (0.001)**	**7 (0.01)**	**8 (0.1)**
**40 – 49**	**44 (0.001)**	**43 (0.001)**	**53 (0.001)**	**56 (0.001)**	**50 (0.001)**	**2 (0.001)**	**7 (0.001)**	**3 (0.001)**	**4 (0.001)**	**9 (0.001)**
**50 – 59**	**38 (0.001)**	**51 (0.001)**	**46 (0.001)**	**54 (0.001)**	**52 (0.001)**	**3 (0.001)**	**10 (0.1)**	**4 (0.001)**	**5 (0.001)**	**15 (0.001)**
**60 – 69**	**41 (0.001)**	**33 (0.05)**	**45 (0.001)**	**55 (0.001)**	**55 (0.001)**	**6 (0.001)**	**5 (0.001)**	**4 (0.001)**	**3 (0.001)**	**17 (0.001)**
**≥ 70**	**50 (0.001)**	**51 (0.1)**	**44 (0.001)**	**54 (0.001)**	**54 (0.001)**	**10 (0.001)**	**5 (0.001)**	**9 (0.001)**	**18 (0.001)**	**18 (0.001)**
**Malay**	**24 (0.001)**	**45 (0.001)**	**23 (0.001)**	**28 (0.001)**	**20 (0.001)**	**1 (0.001)**	**1 (0.1)**	**1 (0.001)**	**2 (0.001)**	**7 (0.001)**
**Chinese**	**11 (0.001)**	**8 (0.05)**	**15 (0.001)**	**14 (0.001)**	**15 (0.001)**	**3 (0.001)**	**8 (0.05)**	**5 (0.001)**	**1 (0.001)**	**2 (0.1)**
**Indian**	**14 (0.001)**	**14 (0.001)**	**14 (0.001)**	**15 (0.001)**	**14 (0.001)**	**2 (0.001)**	**0 (0.05)**	**3 (0.001)**	**2 (0.001)**	**11 (0.1)**
**Others**	**18 (0.001)**	**14 (0.1)**	**18 (0.001)**	**20 (0.001)**	**16 (0.001)**	**0 (0.001)**	**0 (0.05)**	**3 (0.001)**	**8 (0.001)**	**15 (0.001)**
**Urban**	**31 (0.001)**	**33 (0.001)**	**30 (0.001)**	**32 (0.001)**	**31 (0.001)**	**1 (0.001)**	**2 (0.05)**	**2 (0.001)**	**1 (0.001)**	**5 (0.001)**
**Rural**	**35 (0.001)**	**37 (0.001)**	**34 (0.001)**	**38 (0.001)**	**27 (0.001)**	**1 (0.001)**	**4 (0.05)**	**2 (0.001)**	**3 (0.001)**	**12 (0.001)**
**WPM**	**41 (0.001)**	**58 (0.001)**	**51 (0.001)**	**55 (0.001)**	**20 (0.001)**	**3 (0.001)**	**11 (0.01)**	**4 (0.001)**	**6 (0.001)**	**15 (0.001)**
**EPM**	**42 (0.001)**	**57 (0.001)**	**49 (0.001)**	**54 (0.001)**	**23 (0.001)**	**4 (0.001)**	**13 (0.001)**	**4 (0.001)**	**5 (0.001)**	**17 (0.001)**
**EM**	**43 (0.001)**	**25 (0.1)**	**48 (0.001)**	**57 (0.001)**	**24 (0.001)**	**2 (0.001)**	**5 (0.1)**	**4 (0.001)**	**3 (0.001)**	**12 (0.001)**

Note: Figures in parentheses refer to p-values.

Although past studies on Malaysia showed an inverse association between hypertension and education [21,22], our results showed a lack of association between them (Table 6), which nonetheless is consistent with some studies on other countries [45,49]. While there was no association between ethnicity and education by ethnicity among men, it was clear and inverse among women. The prevalence of hypertension was highest among other women with technical education (46%, p > 0.1) followed by Indian women with no (44%, p < 0.001), and technical (39%, p < 0.05) and primary (32%, p < 0.001) education. While there was no association between education and hypertension among urban and rural men, it was inverse among urban women. Apart for differences among women with primary education, there were no other major rural–urban differences in the prevalence of hypertension. Urban women with primary education (42%, p < 0.001) followed by urban (35%, p < 0.001) and rural (35%, p < 0.001) women with no education showed the highest prevalence of hypertension. Among the three regions, the relationship between hypertension and education was clear and inverse only among women in WPM and EPM. Men from EM had higher prevalence of hypertension than men from WPM and EPM and in the university, technical and secondary education groups, while men with primary or no education from EPM had higher prevalence of hypertension than men from WPM and EM. Women with technical, secondary, primary and no education in EPM had the highest prevalence of hypertension among women. However, WPM (23%, p < 0.001) and EM (23%, p < 0.001) showed the highest prevalence of hypertension among university educated women.

**Table 6:** Age-adjusted percentage of men and women having hypertension by education, gender, ethnicity and location

**Age/Ethnicity/**	**Men**					**Women**				
**Location/Region**	**University**	**Technical**	**Secondary**	**Primary**	**None**	**University**	**Technical**	**Secondary**	**Primary**	**None**
**30 – 39**	**19 (0.001)**	**17 (0.05)**	**24 (0.001)**	**26 (0.01)**	**43 (0.1)**	**8 (0.001)**	**7 (0.1)**	**19 (0.001)**	**20 (0.001)**	**14 (0.001)**
**40 – 49**	**33 (0.001)**	**43 (0.001)**	**34 (0.001)**	**39 (0.001)**	**46 (0.001)**	**19 (0.001)**	**19 (0.05)**	**33 (0.001)**	**34 (0.001)**	**30 (0.001)**
**50 – 59**	**46 (0.001)**	**37 (0.001)**	**38 (0.001)**	**47 (0.001)**	**42 (0.001)**	**38 (0.001)**	**25 (0.05)**	**41 (0.001)**	**51 (0.001)**	**46 (0.001)**
**60 – 69**	**51 (0.001)**	**67 (0.05)**	**56 (0.001)**	**63 (0.001)**	**56 (0.001)**	**40 (0.001)**	**56 (0.05)**	**56 (0.001)**	**62 (0.001)**	**56 (0.001)**
**≥ 70**	**63 (0.001)**	**65 (0.001)**	**71 (0.001)**	**69 (0.001)**	**69 (0.001)**	**57 (0.05)**	**60 (0.1)**	**59 (0.001)**	**62 (0.001)**	**66 (0.001)**
**Malay**	**22 (0.001)**	**34 (0.001)**	**19 (0.001)**	**37 (0.001)**	**19 (0.001)**	**11 (0.001)**	**6 (0.05)**	**20 (0.001)**	**27 (0.001)**	**42 (0.001)**
**Chinese**	**16 (0.001)**	**23 (0.001)**	**21 (0.001)**	**35 (0.001)**	**14 (0.001)**	**12 (0.001)**	**7 (0.1)**	**26 (0.001)**	**35 (0.001)**	**36 (0.001)**
**Indian**	**15 (0.001)**	**36 (0.05)**	**18 (0.001)**	**20 (0.01)**	**10 (0.1)**	**8 (0.01)**	**39 (0.05)**	**19 (0.001)**	**32 (0.001)**	**44 (30.001)**
**Others**	**15 (0.01)**	**20 (0.001)**	**21 (0.001)**	**24 (0.001)**	**20 (0.001)**	**8 (0.05)**	**46 (0.1)**	**16 (0.001)**	**19 (0.001)**	**30 (0.001)**
**Urban**	**20 (0.001)**	**24 (0.001)**	**19 (0.001)**	**24 (0.001)**	**19 (0.001)**	**11 (0.001)**	**9 (0.001)**	**20 (0.001)**	**42 (0.001)**	**35 (0.001)**
**Rural**	**20 (0.001)**	**22 (0.001)**	**19 (0.001)**	**27 (0.001)**	**19 (0.001)**	**11 (0.001)**	**10 (0.001)**	**21 (0.001)**	**26 (0.001)**	**35 (0.001)**
**WPM**	**35 (0.001)**	**32 (0.001)**	**37 (0.001)**	**48 (0.001)**	**19 (0.001)**	**23 (0.001)**	**22 (0.001)**	**35 (0.001)**	**46 (0.001)**	**49 (0.001)**
**EPM**	**40 (0.001)**	**40 (0.001)**	**49 (0.001)**	**58 (0.001)**	**26 (0.001)**	**17 (0.001)**	**31 (0.05)**	**36 (0.001)**	**54 (0.001)**	**57 (0.001)**
**EM**	**49 (0.001)**	**75 (0.05)**	**50 (0.001)**	**57 (0.001)**	**25 (0.001)**	**23 (0.001)**	**15 (0.1)**	**32 (0.001)**	**47 (0.001)**	**40 (0.001)**

Note: Figures in parentheses refer to p-values.

An important past study showed that the relationship between diabetes and education was inverse among women, but the middle income group showed the highest prevalence among men in Malaysia [22]. However, our results show that there was no association between diabetes and education (Table 7). Also, the association between age and diabetes was only positive among women in the secondary education group. While the relationship between education and diabetes by ethnicity was positive among the Malay, Chinese and Indian women, it was not obvious among other women, and among men in all ethnic groups. The prevalence of diabetes was highest among Indian men with technical education (26%, p < 0.1) followed by Indian women with no education (20%, p < 0.05), and Indian women with primary education (18%, p < 0.01). The relationship between diabetes and education by location was significant among men and women but it was positive in the former and negative in the latter. Urban men had the highest prevalence of diabetes among respondents with primary (12%, p < 0.001) and no (7%, p < 0.001) education. However, rural men had higher prevalence among university, technical and primary categories, and rural women showed higher prevalence in the university, technical and secondary categories, though all the prevalence levels were low. There was no association between education and diabetes among men and women in the three regions. However, EPM had the highest prevalence of diabetes among men in the education categories of secondary and no education, and among women in all categories except for university education. EM (27%, p < 0.001) had the highest prevalence among men with primary education.

**Table 7:** Age-adjusted percentage of men and women having diabetes by education, gender, ethnicity and location

**Age/Ethnicity/**	**Men**					**Women**				
**Location/Region**	**University**	**Technical**	**Secondary**	**Primary**	**None**	**University**	**Technical**	**Secondary**	**Primary**	**None**
**30 – 39**	**15 (0.001)**	**26 (0.05)**	**21 (0.01)**	**23 (0.001)**	**14 (0.1)**	**7 (0.001)**	**14 (0.1)**	**14 (0.001)**	**23 (0.001)**	**11 (0.05)**
**40 – 49**	**15 (0.001)**	**18 (0.001)**	**18 (0.001)**	**18 (0.001)**	**13 (0.01)**	**7 (0.001)**	**7 (0.1)**	**14 (0.001)**	**17 (0.001)**	**14 (0.001)**
**50 – 59**	**14 (0.001)**	**9 (0.1)**	**24 (0.001)**	**21 (0.001)**	**20 (0.001)**	**12 (0.001)**	**5 (0.1)**	**16 (0.001)**	**22 (0.001)**	**14 (0.001)**
**60 – 69**	**21 (0.001)**	**8 (0.1)**	**22 (0.001)**	**20 (0.001)**	**15 (0.001)**	**10 (0.05)**	**11 (0.1)**	**24 (0.001)**	**20 (0.001)**	**19 (0.001)**
**≥ 70**	**13 (0.1)**	**25 (0.1)**	**24 (0.001)**	**22 (0.001)**	**13 (0.001)**	**20 (0.001)**	**23 (0.1)**	**27 (0.05)**	**18 (0.001)**	**18 (0.001)**
**Malay**	**9 (0.001)**	**24 (0.001)**	**10 (0.001)**	**10 (0.001)**	**5 (0.001)**	**5 (0.001)**	**2 (0.1)**	**10 (0.001)**	**11 (0.001)**	**12 (0.001)**
**Chinese**	**6 (0.001)**	**6 (0.001)**	**6 (0.001)**	**7 (0.001)**	**7 (0.05)**	**1 (0.05)**	**5 (0.1)**	**4 (0.001)**	**6 (0.01)**	**8 (0.05)**
**Indian**	**8 (0.01)**	**26 (0.1)**	**15 (0.001)**	**16 (0.05)**	**3 (0.1)**	**4 (0.05)**	**9 (0.1)**	**7 (0.01)**	**18 (0.01)**	**20 (0.05)**
**Others**	**10 (0.05)**	**9 (0.001)**	**10 (0.001)**	**8 (0.001)**	**6 (0.001)**	**6 (0.1)**	**12 (0.1)**	**12 (0.001)**	**11 (0.001)**	**11 (0.001)**
**Urban**	**8 (0.001)**	**10 (0.001)**	**9 (0.001)**	**12 (0.001)**	**7 (0.001)**	**4 (0.001)**	**3 (0.05)**	**9 (0.001)**	**12 (0.001)**	**12 (0.001)**
**Rural**	**9 (0.001)**	**12 (0.001)**	**9 (0.001)**	**9 (0.001)**	**5 (0.001)**	**8 (0.001)**	**5 (0.05)**	**10 (0.001)**	**10 (0.001)**	**11 (0.001)**
**WPM**	**16 (0.001)**	**17 (0.001)**	**20 (0.001)**	**19 (0.001)**	**6 (0.001)**	**8 (0.001)**	**2 (0.1)**	**15 (0.001)**	**20 (0.001)**	**15 (0.001)**
**EPM**	**17 (0.001)**	**17 (0.05)**	**23 (0.001)**	**19 (0.001)**	**8 (0.001)**	**8 (0.001)**	**31 (0.01)**	**18 (0.001)**	**21 (0.001)**	**20 (0.001)**
**EM**	**12 (0.001)**	**13 (0.1)**	**22 (0.001)**	**27 (0.001)**	**3 (0.05)**	**8 (0.001)**	**5 (0.1)**	**15 (0.001)**	**18 (0.001)**	**10 (0.001)**

Note: Figures in parentheses refer to p-values.

There was no clear relationship between hypercholesterolemia, and education and gender (Table 8), which adds to the past literature reporting varied findings from other countries [2-5,9-11,13,39,50]. However, our results show that the relationship between hypercholesterolemia and education by ethnicity was negative among Malay, Chinese and Indian women but positive among Chinese and other men. Also, our study showed that Malay men in all education categories had the highest prevalence of hypercholesterolemia, which is consistent with some findings on Malaysia and Singapore [51,52]. The highest prevalence was found in the technical education group (69%, p < 0.001). Women with no education had the highest prevalence of hypercholesterolemia in all ethnic groups. Malay women (55%, p < 0.001) showed the highest prevalence of hypercholesterolemia followed by Indian women (51%, p < 0.001). The relationship between hypercholesterolemia and education was negative among rural women. Urban women had higher hypercholesterolemia than rural women in all education categories. The highest prevalence of hypercholesterolemia was recorded by urban women with no education (52%, p < 0.001), and in men among the technically educated (41%, p < 0.001). Among the three regions, the relationship between hypercholesterolemia and education was clear only among men (positive) in WPM and women (negative) in EM. EPM showed the highest prevalence of hypercholesterolemia among men in the technical (74%, p < 0.001) and secondary (73%, p < 0.001) education groups, and among women in the no (67%, p < 0.001) education group. EM showed the highest prevalence among men in the university (72%, p < 0.001), primary (75%, p < 0.001) and no education (28%, p < 0.001) groups. WPM showed the highest prevalence among women with technical (60%, p < 0.001) and secondary (72%, p < 0.001) education.

**Table 8:** Age-adjusted percentage of men and women having hypercholesterolemia by education, gender, ethnicity and location

**Age/Ethnicity/**	**Men**					**Women**				
**Location/Region**	**University**	**Technical**	**Secondary**	**Primary**	**None**	**University**	**Technical**	**Secondary**	**Primary**	**None**
**30 – 39**	**74 (0.001)**	**70 (0.001)**	**69 (0.001)**	**57 (0.001)**	**29 (0.001)**	**57 (0.001)**	**36 (0.05)**	**61 (0.001)**	**54 (0.001)**	**51 (0.001)**
**40 – 49**	**72 (0.001)**	**61 (0.001)**	**73 (0.001)**	**65 (0.001)**	**62 (0.1)**	**63 (0.001)**	**67 (0.001)**	**67 (0.001)**	**61 (0.001)**	**57 (0.001)**
**50 – 59**	**77 (0.001)**	**77 (0.001)**	**72 (0.001)**	**67 (0.001)**	**52 (0.001)**	**76 (0.001)**	**70 (0.001)**	**77 (0.001)**	**76 (0.001)**	**62 (0.001)**
**60 – 69**	**61 (0.001)**	**67 (0.01)**	**69 (0.001)**	**71 (0.001)**	**54 (0.001)**	**82 (0.001)**	**78 (0.01)**	**74 (0.001)**	**77 (0.001)**	**72 (0.001)**
**≥ 70**	**38 (0.05)**	**65 (0.1)**	**73 (0.001)**	**70 (0.001)**	**52 (0.001)**	**57 (0.05)**	**60 (0.001)**	**82 (0.001)**	**80 (0.001)**	**70 (0.001)**
**Malay**	**38 (0.001)**	**69 (0.001)**	**33 (0.001)**	**36 (0.001)**	**22 (0.001)**	**33 (0.001)**	**16 (0.001)**	**41 (0.001)**	**41 (0.001)**	**55 (0.001)**
**Chinese**	**32 (0.001)**	**23 (0.001)**	**26 (0.001)**	**24 (0.001)**	**15 (0.001)**	**33 (0.001)**	**41 (0.001)**	**39 (0.001)**	**41 (0.001)**	**42 (0.001)**
**Indian**	**27 (0.001)**	**34 (0.01)**	**22 (0.001)**	**27 (0.001)**	**17 (0.01)**	**33 (0.001)**	**39 (0.05)**	**34 (0.001)**	**35 (0.001)**	**51 (0.001)**
**Others**	**34 (0.001)**	**25 (0.001)**	**23 (0.001)**	**20 (0.001)**	**13 (0.001)**	**15 (0.01)**	**14 (0.1)**	**27 (0.001)**	**26 (0.001)**	**33 (0.001)**
**Urban**	**36 (0.001)**	**41 (0.001)**	**31 (0.001)**	**32 (0.001)**	**23 (0.001)**	**32 (0.001)**	**24 (0.001)**	**42 (0.001)**	**41 (0.001)**	**52 (0.001)**
**Rural**	**39 (0.001)**	**40 (0.001)**	**31 (0.001)**	**32 (0.001)**	**16 (0.001)**	**32 (0.001)**	**15 (0.001)**	**37 (0.001)**	**37 (0.001)**	**43 (0.001)**
**WPM**	**71 (0.001)**	**63 (0.001)**	**71 (0.001)**	**64 (0.001)**	**21 (0.001)**	**67 (0.001)**	**60 (0.001)**	**72 (0.001)**	**70 (0.001)**	**65 (0.001)**
**EPM**	**71 (0.001)**	**74 (0.001)**	**73 (0.001)**	**70 (0.001)**	**21 (0.001)**	**63 (0.001)**	**56 (0.01)**	**67 (0.001)**	**72 (0.001)**	**67 (0.001)**
**EM**	**72 (0.001)**	**50 (0.001)**	**69 (0.001)**	**75 (0.001)**	**28 (0.001)**	**64 (0.001)**	**55 (0.001)**	**66 (0.001)**	**74 (0.001)**	**65 (0.001)**

Note: Figures in parentheses refer to p-values.

The relationship between education and being overweight among men was generally inverse (Table 9), which supports most findings on Malaysia [25,26,28,29]. However, our results show no clear relationship between education levels and being overweight among women. The relationship between being overweight and education by ethnicity was clear and positive among Malay and Chinese men, while it was negative among Indian women. In addition, except for primary education, Malays had the highest prevalence of being overweight among men, which largely supports past results on Singapore and Malaysia [26,51]. However, Indians with primary education had the highest prevalence of being overweight among men (20%, p < 0.001). Also, except for university and secondary education, Indians had the highest prevalence of being overweight among women. Malays had the highest prevalence of being overweight among university (19%, p < 0.001) and secondary (28%, p < 0.001) educated women. Whereas the relationship between being overweight and education was positive among urban and rural men, it was not clear among women. Urban men in the secondary (15%, p < 0.001), primary (14%, 0.001) and no education (9%, p < 0.001) groups showed higher prevalence of being overweight than rural men in the same education categories respectively. Except for the technically educated, urban women showed higher prevalence of being overweight than rural women in all other education groups. Among the three regions, the relationship between being overweight and education was clear and positive only in WPM. Also, WPM showed the highest prevalence among the university educated (37%, p < 0.001), while EM showed the highest prevalence in the secondary (41%, p < 0.001) and primary (28%, p < 0.001) education groups among men. EPM showed the highest prevalence among men with no education (7%, p < 0.001), though the levels were low in all three regions. Whereas WPM showed the highest prevalence among women in the university (35%, p < 0.001) and secondary (42%, p < 0.001) education groups, EM showed the highest prevalence among women in the technical (20%, p < 0.001) and primary (43%, p < 0.001) education groups.

**Table 9:** Age-adjusted percentage of men and women being overweight by education level, gender, age, ethnicity and location

**Age/Ethnicity/**	**Men**					**Women**				
**Location/Region**	**University**	**Technical**	**Secondary**	**Primary**	**None**	**University**	**Technical**	**Secondary**	**Primary**	**None**
**30 – 39**	**40 (0.001)**	**30 (0.01)**	**31 (0.001)**	**23 (0.001)**	**14 ( 0.1)**	**29 (0.001)**	**14 (0.001)**	**34 (0.001)**	**27 (0.001)**	**19 (0.01)**
**40 – 49**	**38 (0.001)**	**47 (0.001)**	**34 (0.001)**	**31 (0.001)**	**19 (0.001)**	**34 (0.001)**	**26 (0.001)**	**43 (0.001)**	**41 (0.001)**	**27 (0.001)**
**50 – 59**	**35 (0.001)**	**40 (0.001)**	**38 (0.001)**	**27 (0.001)**	**20 (0.001)**	**39 (0.001)**	**20 (0.001)**	**45 (0.001)**	**44 (0.001)**	**28 (0.001)**
**60 – 69**	**29 (0.001)**	**17 (0.1)**	**32 (0.001)**	**26 (0.001)**	**12 (0.001)**	**40 (0.001)**	**11 (0.001)**	**44 (0.001)**	**43 (0.001)**	**21 (0.001)**
**≥ 70**	**25 (0.05)**	**20 (0.001)**	**26 (0.001)**	**16 (0.001)**	**11 (0.001)**	**29 (0.001)**	**30 (0.001)**	**32 (0.01)**	**31 (0.001)**	**11 (0.001)**
**Malay**	**21 (0.001)**	**27 (0.001)**	**16 (0.001)**	**13 (0.001)**	**6 (0.001)**	**19 (0.001)**	**9 (0.001)**	**28 (0.001)**	**24 (0.001)**	**18 (0.001)**
**Chinese**	**9 (0.001)**	**11 ( 0.05 )**	**9 (0.001)**	**7 (0.001)**	**2 ( 0.1)**	**6 (0.001)**	**10 (0.001)**	**9 (0.001)**	**15 (0.001)**	**14 (0.001)**
**Indian**	**13 (0.001)**	**16 ( 0.1)**	**15 (0.001)**	**20 (0.001)**	**8 ( 0.1)**	**17 (0.001)**	**20 (0.001)**	**17 (0.001)**	**34 (0.001)**	**34 (0.01)**
**Others**	**13 (0.05)**	**14 ( 0.1)**	**15 (0.001)**	**9 (0.001)**	**4 (0.001)**	**12 (0.001)**	**14 (0.001)**	**13 (0.001)**	**12 (0.001)**	**9 (0.001)**
**Urban**	**18 (0.001)**	**22 (0.001)**	**15 (0.001)**	**14 (0.001)**	**9 (0.001)**	**17 (0.001)**	**6 (0.001)**	**24 (0.001)**	**24 (0.001)**	**20 (0.001)**
**Rural**	**18 (0.001)**	**23 (0.001)**	**14 (0.001)**	**11 (0.001)**	**3 (0.001)**	**14 (0.001)**	**10 (0.001)**	**23 (0.001)**	**21 (0.001)**	**12 (0.001)**
**WPM**	**37 (0.001)**	**38 (0.001)**	**34 (0.001)**	**26 (0.001)**	**5 (0.001)**	**35 (0.001)**	**18 (0.001)**	**42 (0.001)**	**41 (0.001)**	**22 (0.001)**
**EPM**	**33 (0.001)**	**31 (0.001)**	**34 (0.001)**	**24 (0.001)**	**7 (0.001)**	**29 (0.001)**	**19 (0.001)**	**41 (0.001)**	**40 (0.001)**	**18 (0.001)**
**EM**	**34 (0.001)**	**38 ( 0.1)**	**41 (0.001)**	**28 (0.001)**	**5 (0.001)**	**31 (0.001)**	**20 (0.001)**	**41 (0.001)**	**43 (0.001)**	**29 (0.001)**

Note: Figures in parentheses refer to p-values.

### Income

There was a positive relationship between diabetes, hypercholesterolemia and being overweight, and income among men (Table 10). These results differ from one study on Malaysia in which the relationship between diabetes and income was inverse, while the middle income had the highest prevalence of hypercholesterolemia [22]. Also, our results show that the relationship between hypertension and hypercholesterolemia, and income was negative among women, which is consistent with the findings from similar studies on Malaysia and the developed countries [11,13,22]. The relationship between income and diabetes was positive among women. Our results did not corroborate with some studies on Malaysia that showed middle income women having the lowest prevalence of diabetes, hypertension, while high income women the lowest prevalence of hypercholesterolemia [22-24].

**Table 10:** Age-adjusted percentage of men and women having CVD risk factors by income level

**CVD risk factors**	**Men**			**Women**		
	**Low**	**Middle**	**High**	**Low**	**Middle**	**High**
Hypertension*	**52 (0.001)**	**47 (0.001)**	**58 (0.001)**	**51 (0.001)**	**37 (0.001)**	**29 (0.001)**
**Diabetes+**	**15 (0.001)**	**19 (0.001)**	**22 (0.001)**	**16 (0.001)**	**11 (0.001)**	**9 (0.001)**
**Hypercholesterolemiaδ**	**62 (0.001)**	**73 (0.001)**	**78 (0.001)**	**73 (0.001)**	**73 (0.001)**	**68 (0.001)**
**Smokerθ**	**51 (0.001)**	**48 (0.001)**	**55 (0.001)**	**9 (0.001)**	**3 (0.001)**	**5 (0.001)**
**Overweightψ**	**21 (0.001)**	**38 (0.001)**	**49 (0.001)**	**35 (0.001)**	**41 (0.001)**	**32 (0.001)**

Note: Figures in parentheses refer to p-values.

*Mean *SBP* (Systolic Blood Pressure) ≥ 140 mm Hg or Mean *DBP* (Diastolic Blood Pressure) ≥ 90 mm Hg or using medication.

**+**Mean plasma glucose ≥ 126 mg/dL or 7.0 mmol/Lit.

δMean Plasma cholesterol ≥ 5.2 mmol/Lit.

θSmokes at least one cigarette per day.

ψ*BMI* (Body Mass Index) ≥ 25.0.

Our results showed that more men than women smoked in all income, age, ethnic and location categories (Table 11). Except for the age categories of 50–59 and 70 and above, the relationship between income and smoking was inverse among men, which supports some studies on Malaysia [22-24]. The relationship between smoking and income among men was positive in the 50–59 and 60–69 age groups. However, the relationship was inverse among women in the age groups of 30–39 and 50–59. Malays had the highest prevalence of smoking among men. The relationship between smoking and income in men was negative among Malays, Chinese and others. In women, the prevalence of smoking was highest among the others in the low income (15%, p < 0.001) and high income (10%, p < 0.001) groups, and Chinese among the middle income (12%, p < 0.001) groups. More rural men smoked than urban men in all income categories. Also, rural women (9%, p < 0.001) smoked more than urban women (7%, p < 0.001) in the low income group, while the prevalence of smoking was similar between urban and rural women among the middle and high income groups. There was no association between smoking and income between the three regions. However, EM had the highest prevalence of smoking among men in the low (43%, p < 0.001) and high (56%, p < 0.001) income groups, while EPM had the highest prevalence of smoking among women in the low (14%, p < 0.001) income groups.

**Table 11:** Age-adjusted percentage of men and women smoking by income level, gender, age, ethnicity and location

**Age/Ethnicity/**	**Men**			**Women**		
**Location/Region**	**Low**	**Middle**	**High**	**Low**	**Middle**	**High**
**30 – 39**	**60 (0.001)**	**57 (0.001)**	**53 (0.001)**	**6 (0.001)**	**4 (0.01)**	**2 (0.1)**
**40 – 49**	**57 (0.001)**	**49 (0.001)**	**41 (0.001)**	**5 (0.001)**	**3 (0.001)**	**5 (0.001)**
**50 – 59**	**43 (0.001)**	**46 (0.001)**	**47 (0.001)**	**9 (0.001)**	**3 (0.01)**	**2 (0.1)**
**60 – 69**	**52 (0.001)**	**49 (0.001)**	**38 (0.001)**	**10 (0.001)**	**2 (0.1)**	**16 (0.05)**
**≥ 70**	**56 (0.001)**	**35 (0.01)**	**45 (0.1)**	**14 (0.001)**	**1 (0.001)**	**2 (0.001)**
**Malay**	**58 (0.001)**	**51 (0.001)**	**48 (0.001)**	**5 (0.001)**	**2 (0.001)**	**4 (0.001)**
**Chinese**	**42 (0.001)**	**31 (0.001)**	**10 (0.001)**	**4 (0.05)**	**12 (0.001)**	**6 (0.05)**
**Indian**	**11 (0.1)**	**34 (0.001)**	**29 (0.05)**	**2 (0.001)**	**3 (0.1)**	**4 (0.1)**
**Others**	**43 (0.001)**	**40 (0.001)**	**13 (0.1)**	**15 (0.001)**	**6 (0.001)**	**10 (0.001)**
**Urban**	**37 (0.001)**	**37 (0.001)**	**30 (0.001)**	**7 (0.001)**	**3 (0.001)**	**4 (0.001)**
**Rural**	**40 (0.001)**	**38 (0.001)**	**39 (0.001)**	**9 (0.001)**	**3 (0.001)**	**4 (0.001)**
**WPM**	**41 (0.001)**	**49 (0.001)**	**53 (0.001)**	**4 (0.001)**	**3 (0.001)**	**8 (0.001)**
**EPM**	**40 (0.001)**	**48 (0.001)**	**54 (0.001)**	**14 (0.001)**	**3 (0.05)**	**8 (0.01)**
**EM**	**43 (0.001)**	**49 (0.001)**	**56 (0.001)**	**2 (0.001)**	**4 (0.001)**	**6 (0.1)**

Figures in parentheses refer to p-value.

The relationship between hypertension and income by gender was negative in the age category of 40–49 but was positive in the age categories of 50–59 and ≥70 years among men (Table 12), which we could not compare with past studies on Malaysia because of the use of different age intervals [21,22]. This association was inverse among women in all but the category of ≥70 years. The relationship between hypertension and income by ethnicity was inverse among Indian men and women, Chinese men and Malay women. However, low income Indian women (70%, p < 0.05) and men (56%, p < 0.05) showed the highest prevalence of hypertension. Men (53%, p < 0.001) and women (41%, p < 0.001) in the others category showed the highest prevalence of hypertension among the middle income. Malay men (38%, p < 0.001) and other women (40%, p > 0.1) showed the highest prevalence of hypertension among the high income group. Whereas the relationship between income and hypertension was inverse among urban men and women, it was only clear and inverse among rural men. Rural men and women showed higher prevalence of hypertension than urban men and women in all income groups. The relationship between hypertension and income was positive in WPM and EM among men, and in all three regions among women. Whereas EPM showed the highest prevalence of hypertension among women in all income categories, EPM showed the highest prevalence of hypertension among low income men. EM had the highest prevalence among middle and high income men.

**Table 12:** Age-adjusted percentage of men and women having hypertension by income level, gender, age, ethnicity and location

**Age/Ethnicity/**	**Men**			**Women**		
**Location/Region**	**Low**	**Middle**	**High**	**Low**	**Middle**	**High**
**30 – 39**	**20 (0.001)**	**27 (0.001)**	**18 (0.01)**	**20 (0.001)**	**16 (0.001)**	**8 (0.01)**
**40 – 49**	**38 (0.001)**	**33 (0.001)**	**33 (0.001)**	**35 (0.001)**	**27 (0.001)**	**22 (0.001)**
**50 – 59**	**39 (0.001)**	**45 (0.001)**	**50 (0.001)**	**57 (0.001)**	**44 (0.001)**	**32 (0.001)**
**60 – 69**	**60 (0.001)**	**64 (0.001)**	**57 (0.001)**	**57 (0.001)**	**57 (0.001)**	**56 (0.001)**
**≥ 70**	**68 (0.001)**	**88 (0.001)**	**89 (0.1)**	**66 (0.001)**	**71 (0.05)**	**76 (0.001)**
**Malay**	**45 (0.001)**	**37 (0.001)**	**38 (0.001)**	**44 (0.001)**	**33 (0.001)**	**26 (0.001)**
**Chinese**	**50 (0.001)**	**43 (0.001)**	**27 (0.001)**	**43 (0.01)**	**22 (0.001)**	**26 (0.001)**
**Indian**	**56 (0.05)**	**32 (0.001)**	**32 (0.01)**	**70 (0.05)**	**35 (0.001)**	**19 (0.01)**
**Others**	**51 (0.001)**	**53 (0.001)**	**13 (0.1)**	**40 (0.001)**	**41 (0.001)**	**40 (0.1)**
**Urban**	**48 (0.001)**	**39 (0.001)**	**39 (0.001)**	**39 (0.001)**	**29 (0.001)**	**25 (0.001)**
**Rural**	**49 (0.001)**	**46 (0.001)**	**44 (0.001)**	**44 (0.001)**	**40 (0.001)**	**42 (0.001)**
**WPM**	**36 (0.001)**	**37 (0.001)**	**44 (0.001)**	**24 (0.001)**	**30 (0.001)**	**40 (0.001)**
**EPM**	**47 (0.001)**	**46 (0.001)**	**55 (0.001)**	**28 (0.001)**	**41 (0.001)**	**49 (0.001)**
**EM**	**43 (0.001)**	**54 (0.001)**	**57 (0.001)**	**27 (0.001)**	**32 (0.001)**	**42 (0.001)**

Figures in parentheses refer to p-value.

While one study showed an inverse relationship between diabetes and income among men, and the middle income showing the lowest prevalence among women in Malaysia [22], our study showed that the association between diabetes and income was positive in the age groups of 50–59 and 60–69, negative in the age group of ≥70 (Table 13). However, the relationship between diabetes and income was negative among women in the age groups of 40–49, 50–59 and ≥70. The relationship between income and diabetes by ethnicity was inverse among women but was not clear among men in all ethnic groups. Indian women (18%, p < 0.001) and Malay men (16%, p < 0.001) had the highest prevalence of diabetes among the low income. Indian men (29%, p < 0.001) and other women (17%, p < 0.01) had the highest prevalence of diabetes among the middle income. Chinese men (18%, p < 0.001) and other women (20%, p > 0.1) had the highest prevalence of diabetes among the high income. The relationship between diabetes and income was positive among rural men and women, and negative among urban women. The relationship between diabetes and income was negative in rural women, while it was positive in urban women. The relationship between income and diabetes was positive among women but it was not obvious among men in all the three regions. EPM showed the highest prevalence among low (14%, p < 0.001) and middle (14%, p < 0.001) income women, and among middle income men (21%, p < 0.001). EM showed the highest prevalence among low income (22%, p < 0.001) men.

**Table 13:** Age-adjusted percentage of men and women having diabetes by income level, gender, age, ethnicity and location

**Age/Ethnicity/**	**Men**			**Women**		
**Location/Region**	**Low**	**Middle**	**High**	**Low**	**Middle**	**High**
**30 – 39**	**11 (0.001)**	**23 (0.001)**	**13 (0.01)**	**8 (0.001)**	**11 (0.001)**	**7 (0.01)**
**40 – 49**	**14 (0.001)**	**18 (0.001)**	**16 (0.001)**	**14 (0.001)**	**8 (0.001)**	**6 (0.001)**
**50 – 59**	**13 (0.001)**	**20 (0.001)**	**38 (0.001)**	**20 (0.001)**	**15 (0.001)**	**10 (0.001)**
**60 – 69**	**17 (0.001)**	**18 (0.001)**	**19 (0.01)**	**16 (0.001)**	**14 (0.001)**	**20 (0.01)**
**≥ 70**	**13 (0.001)**	**12 (0.1)**	**11 (0.001)**	**15 (0.001)**	**8 (0.001)**	**1 (0.001)**
**Malay**	**16 (0.001)**	**20 (0.001)**	**17 (0.001)**	**16 (0.001)**	**11 (0.001)**	**8 (0.001)**
**Chinese**	**12 (0.1)**	**5 (0.01)**	**18 (0.001)**	**4 (0.1)**	**4 (0.01)**	**6 (0.01)**
**Indian**	**11 (0.1)**	**29 (0.001)**	**11 (0.1)**	**18 (0.001)**	**14 (0.01)**	**12 (0.1)**
**Others**	**7 (0.001)**	**18 (0.1)**	**12 (0.001)**	**11 (0.001)**	**17 (0.01)**	**20 (0.1)**
**Urban**	**20 (0.001)**	**21 (0.001)**	**18 (0.001)**	**18 (0.001)**	**9 (0.001)**	**8 (0.001)**
**Rural**	**14 (0.001)**	**17 (0.001)**	**25 (0.001)**	**14 (0.001)**	**15 (0.001)**	**15 (0.001)**
**WPM**	**15 (0.001)**	**19 (0.001)**	**15 (0.001)**	**7 (0.001)**	**9 (0.001)**	**14 (0.001)**
**EPM**	**17 (0.001)**	**21 (0.001)**	**13 (0.001)**	**14 (0.001)**	**14 (0.001)**	**19 (0.01)**
**EM**	**22 (0.001)**	**17 (0.001)**	**19 (0.001)**	**9 (0.001)**	**13 (0.001)**	**19 (0.001)**

Figures in parentheses refer to p-value.

Although there was no obvious relationship reported between hypercholesterolemia and income by one study on Malaysia [22], our study showed that the prevalence of hypercholesterolemia increased with income in the age categories of 30–39, 50–59, 60–69 and ≥70 years among men, and ≥70 among women (Table 14)). Hypercholesterolemia increased with age among middle income men and women. However, the relationship between hypercholesterolemia and income by ethnicity was only obvious and positive among Chinese and other men, and negative among Malay, Indian and other women. Indian women (82%, p < 0.001) and men (78%, p < 0.001) showed the highest prevalence of hypercholesterolemia among the low income. Malay men (67%, p < 0.001) and women (73%, p < 0.001) showed the highest prevalence of hypercholesterolemia among the middle income. Other men (88%, p < 0.001) and Malay women (67%, p < 0.001) showed the highest prevalence of hypercholesterolemia among the high income. There was no association between hypercholesterolemia and income among urban and rural women, but it was negative among urban and rural men. Among the three regions, the relationship between hypercholesterolemia and income was clear and positive only among men in WPM while it was negative among women in EM. However, EPM showed the highest prevalence among the low (77%, p < 0.001) and middle (76%, p < 0.001) income groups. Among women, WPM and EPM had the highest prevalence in the middle (71%, p < 0.001) and high (67%, p < 0.001) income groups. EM showed the highest prevalence among low income men (73%, p < 0.001) and women (74%, p < 0.001).

**Table 14:** Age-adjusted percentage of men and women having hypercholesterolemia by income level, gender, age, ethnicity and location

**Age/Ethnicity/**	**Men**			**Women**		
**Location/Region**	**Low**	**Middle**	**High**	**Low**	**Middle**	**High**
**30 – 39**	**49 (0.001)**	**75 (0.001)**	**76 (0.001)**	**53 (0.001)**	**60 (0.001)**	**51 (0.001)**
**40 – 49**	**66 (0.001)**	**75 (0.001)**	**73 (0.001)**	**61 (0.001)**	**69 (0.001)**	**62 (0.001)**
**50 – 59**	**54 (0.001)**	**75 (0.001)**	**86 (0.001)**	**87 (0.001)**	**76 (0.001)**	**76 (0.001)**
**60 – 69**	**66 (0.001)**	**67 (0.001)**	**73 (0.001)**	**73 (0.001)**	**81 (0.001)**	**68 (0.001)**
**≥ 70**	**61 (0.001)**	**76 (0.001)**	**80 (0.001)**	**70 (0.001)**	**86 (0.01)**	**88 (0.001)**
**Malay**	**63 (0.001)**	**67 (0.001)**	**66 (0.001)**	**75 (0.001)**	**73 (0.001)**	**67 (0.001)**
**Chinese**	**54 (0.001)**	**64 (0.001)**	**68 (0.001)**	**65 (0.001)**	**63 (0.001)**	**66 (0.001)**
**Indian**	**78 (0.01)**	**58 (0.001)**	**61 (0.001)**	**82 (0.001)**	**65 (0.001)**	**62 (0.001)**
**Others**	**37 (0.001)**	**56 (0.001)**	**88 (0.01)**	**46 (0.001)**	**46 (0.001)**	**20 (0.1)**
**Urban**	**76 (0.001)**	**74 (0.001)**	**73 (0.001)**	**75 (0.001)**	**72 (0.001)**	**54 (0.001)**
**Rural**	**61 (0.001)**	**74 (0.001)**	**81 (0.001)**	**65 (0.001)**	**67 (0.001)**	**62 (0.001)**
**WPM**	**73 (0.001)**	**74 (0.001)**	**62 (0.001)**	**63 (0.001)**	**71 (0.001)**	**67 (0.001)**
**EPM**	**77 (0.001)**	**76 (0.001)**	**65 (0.001)**	**51 (0.001)**	**71 (0.001)**	**67 (0.001)**
**EM**	**73 (0.001)**	**69 (0.001)**	**71 (0.001)**	**74 (0.001)**	**69 (0.001)**	**66 (0.001)**

Figures in parentheses refer to p-value.

The relationship between being overweight and income was positive among men in the age category of ≥70 (Table 15), which does not support some of the findings on Malaysia [25,26,28,52]. However, the relationship between being overweight and income among women was negative in the age categories of 50–59 and ≥70. The association between being overweight and income by ethnicity was positive among Malay and other men, while it was negative among Chinese women. Chinese men (27%, p < 0.01) had the highest prevalence of being overweight among the low income, while Malay men had the highest prevalence of being overweight among the middle (40%, p < 0.001) and high (43%, p < 0.001) income. Malay women had the highest prevalence of being overweight in all income groups. The relationship between being overweight and income by location was clear only among women: it was negative among urban women and positive among rural women. Urban men showed higher prevalence of being overweight than rural men in all income categories. Whereas urban women showed higher prevalence of being overweight than rural women in the low and middle income groups, it was the reverse in the high income group. Among the three regions, there was a clear and negative association between being overweight and income only among women in EPM. Also, EPM showed the highest prevalence among low (39%, p < 0.001) and middle income (42%, p < 0.01) men. EM had the highest prevalence among high income (29%, p < 0.001) men. The results were mixed among women, with EPM, WPM and EM showing the highest prevalence among the low, middle and high income groups respectively.

**Table 15:** Age-adjusted percentage of men and women being overweight by income level, gender, age, ethnicity and location

**Age/Ethnicity/**	**Men**			**Women**		
**Location/Region**	**Low**	**Middle**	**High**	**Low**	**Middle**	**High**
**30 – 39**	**28 (0.001)**	**34 (0.001)**	**29 (0.001)**	**27 (0.001)**	**32 (0.001)**	**27 (0.001)**
**40 – 49**	**24 (0.001)**	**42 (0.001)**	**37 (0.001)**	**39 (0.001)**	**43 (0.001)**	**33 (0.001)**
**50 – 59**	**24 (0.001)**	**40 (0.001)**	**39 (0.001)**	**45 (0.001)**	**43 (0.001)**	**31 (0.001)**
**60 – 69**	**22 (0.001)**	**37 (0.001)**	**30 (0.001)**	**33 (0.001)**	**42 (0.001)**	**36 (0.01)**
**≥ 70**	**14 (0.001)**	**18 (0.1)**	**33 (0.1)**	**15 (0.001)**	**29 (0.1)**	**31 (0.001)**
**Malay**	**26 (0.001)**	**40 (0.001)**	**43 (0.001)**	**42 (0.001)**	**36 (0.001)**	**37 (0.001)**
**Chinese**	**27 (0.01)**	**29 (0.001)**	**19 (0.001)**	**13 (0.1)**	**12 (0.001)**	**9 (0.01)**
**Indian**	**22 (0.1)**	**34 (0.001)**	**21 (0.01)**	**18 (0.1)**	**35 (0.001)**	**31 (0.01)**
**Others**	**17 (0.001)**	**37 (0.001)**	**38 (0.1)**	**18 (0.001)**	**26 (0.001)**	**20 (0.001)**
**Urban**	**32 (0.001)**	**39 (0.001)**	**38 (0.001)**	**45 (0.001)**	**42 (0.001)**	**31 (0.001)**
**Rural**	**22 (0.001)**	**39 (0.001)**	**31 (0.001)**	**32 (0.001)**	**39 (0.001)**	**42 (0.001)**
**WPM**	**37 (0.001)**	**38 (0.001)**	**23 (0.001)**	**32 (0.001)**	**42 (0.001)**	**36 (0.001)**
**EPM**	**39 (0.001)**	**42 (0.001)**	**23 (0.001)**	**40 (0.001)**	**39 (0.001)**	**31 (0.001)**
**EM**	**35 (0.001)**	**39 (0.001)**	**29 (0.001)**	**29 (0.001)**	**40 (0.001)**	**37 (0.001)**

Figures in parentheses refer to p-value.

## Conclusions

The results generally tallied with the findings from the developed countries with smoking where men consistently smoked more than women in all education and income categories [9-11]. Smoking and hypertension showed an inverse relationship with education among men and women. However, while income showed a negative relationship with hypertension among women, it did not have an association with hypertension among men, and with smoking among both men and women. Also, the relationship between income, and diabetes and hypercholesterolemia was positive among men, while it was negative among women. There was also a positive association between income and being overweight among men. In addition, the relationship between the CVD risk factors, and education and income varied by ethnicity, age and location. Hence, policy makers and administrators should take account of the CVD risk factors by socioeconomic, demographic and geographic characteristics of the population in the country when devising programmes to reduce deaths caused by CVD-related diseases.

This study has some limitations that future studies should avoid. Firstly, the use of education and income provide only a crude estimate of SES. Secondly, it will be good to find local consumer prices to adjust for inflation effects on income. Thirdly, panel data, which refers to data collected from multiple but the same respondents over time, is superior when analysing causal factors [53].

Nevertheless, the prevalence of CVD risk factors between high and low levels of SES is still obvious, and the results show a need to address them when formulating preventive programmes.

## Abbreviations

CVD: Cardiovascular disease; WPM: Western Peninsular Malaysia; EPM: Eastern Peninsular Malaysia; EM: East Malaysia; SES: Socioeconomic status; MYR: Malaysian Ringgit; PURE: Prospective Urban Rural Epidemiology; REDISCOVER: Responding to Increasing Cardiovascular Disease Prevalence; SEE: Socioeconomic epidemiology; LRGS: Long-term Research Grant Scheme.

## Competing interests

The authors declare that they have no competing interests.

## Authors’ contributions

RR: Is the head of the socioeconomic epidemiology (SEE) component of the REDISCOVER Project. He conceived the paper, the literature review, the methodology to be used, directed the processing of data and statistical analysis, and took charge of the writing of the paper, as well as, the final revisions. YK: Is the head of the REDISCOVER Project, which is a component of the PURE project. He conceived the project, defined the parameters of the cardiovascular risk factors, obtained approval from the ethics committee and coordinated the collection of the data, and commented on the paper. AM: Is a research assistant with the SEE. He undertook the age adjustment and statistical analysis used in the paper. RM: Is a member of SEE. He contributed to the literature review used in the paper, and commented on the paper. TM: Is a member with SEE. He assisted with the logistics involved in the project, and commented on the drafts. SKC: Is a member of SEE. He commented on the drafts. KS: Is a research assistant with the SEE. She assisted with the statistical analysis used in the paper. All authors read and approved the final manuscript. ABN: Is a research assistant with the REDISCOVER Project. She coordinated the collection and processing of the data used in the paper.

## Pre-publication history

The pre-publication history for this paper can be accessed here:

http://www.biomedcentral.com/1471-2458/13/886/prepub
